# A Wavefront Division Polarimeter for the Measurements of Solute Concentrations in Solutions

**DOI:** 10.3390/s17122844

**Published:** 2017-12-08

**Authors:** Sergio Calixto, Geminiano Martinez-Ponce, Guillermo Garnica, Susana Figueroa-Gerstenmaier

**Affiliations:** 1 Centro de Investigaciones en Optica, Loma del Bosque 115, Leon 37150, Mexico; geminis@cio.mx (G.M.-P.); garnica@cio.mx (G.G.); 2 Departamento de Ingenierias Quimica, Electrónica y Biomedica, Division de Ciencias e Ingenierias, Universidad de Guanajuato Campus Leon, Loma del bosque 103, Leon 37150, Mexico; sfigueroa@ugto.mx

**Keywords:** polarimeter, interferometer, solutions concentrations, refractive index, specific rotation.

## Abstract

Polarimeters are useful instruments that measure concentrations of optically active substances in a given solution. The conventional polarimetric principle consists of measuring the rotation angle of linearly polarized light. Here, we present a novel polarimeter based on the study of interference patterns. A Mach–Zehnder interferometer with linearly polarized light at the input is used. One beam passes through the liquid sample and the other is a reference beam. As the linearly polarized sample beam propagates through the optically active solution the vibration plane of the electric field will rotate. As a result, the visibility of the interference pattern at the interferometer output will decrease. Fringe contrast will be maximum when both beams present a polarization perpendicular to the plane of incidence. However, minimum visibility is obtained when, after propagation through the sample the polarization of the sample beam is oriented parallel to the plane of incidence. By using different solute concentrations, a calibration plot is obtained showing the behavior of visibility.

## 1. Introduction

A substance is said to be optically active [1] if it rotates the vibration plane of linearly polarized light. The rotation angle depends, among other parameters, on the substance concentration and the optical path length. This optical property has been industrially used for a long time as a reference to quality control. Sugars [2] and essential oils [3] are among the substances that are measured using optical properties. The specific rotatory power of a substance is modeled through Biot’s law [4]:

(1) $${\left(\alpha \right)}_{\lambda }^{T}=\alpha /\mathrm{lc}$$

where α is the rotation angle, l is the optical path length and c is the substance concentration. Solution temperature T and used wavelength λ are also related with the observed rotatory power. Instruments that measure the rotation of the plane of vibration produced by an optically active solution are known as polarimeters. In a basic configuration a polarimeter consists of: (a) a light source giving linearly polarized light with known orientation, or a light source with a fixed linear polarizer, (b) sample tubes of regularized length, where the liquid tested is introduced, and (c) an analyzer fixed in a graduated rotatory mount to measure the rotation angle following the null method principle. Polarizers could be of the sheet-type dichroic or polarizing (Nicol) prisms to mention but two.

One type of polarimeters is based on the half-shadow angle phenomenon [5,6]. Polarimeters such as the biquartz, Laurent half wave plate, the Cornu-Jellet prism, Lippich polarizer and others are based on the half-shadow angle. Some of them can be used with white light but others with monochromatic light. Lippich instruments could show an accuracy of 0.005°.

The polarimeters that we have mentioned in the last paragraph depend on a time sequential activity. That is, they take measurements one at a time. To avoid this time dependence polarimeters based on the wavefront division have been suggested [5]. They can measure the Stokes parameters. Different parts of the wavefront are analyzed with separate polarization elements and detectors. Among these wavefront division polarimeters are the four-channel polarimeter and the Azzam’s four detector photomultiplier. Other wavefront division polarimeters are based on gratings [5,7] and on the wavefront division by parallel plane glass plates [5].

In this article we suggest a new method, to our knowledge, to measure the concentration of an optically active substance based on a two beam interference pattern. When the linear polarization of two interference beams is parallel to each other, and perpendicular to the plane of incidence, the visibility of the fringes is maximum. As the plane of polarization of one beam rotates, due to the pass of light through an active medium, the visibility of the fringes decreases. The amount of rotation is a function of the solution concentration and the length of the optical path. The interference patterns are recorded with a CCD camera linked to a computer. Then it is possible to do the calculations and infer visibility. In Section 2 we describe briefly optical activity and some details of the materials used in the experiments. Also, an optical characterization regarding material’s refractive index is presented. In Section 3 is described the interferometric optical configuration. Section 4 shows the results including the calibration plots, one for each material tested. Finally, in Section 5 we conclude.

## 2. Optical Activity

Usually when linearly polarized light passes through liquids its plane of polarization is unaffected [1]. However, there are some liquids that show optical activity, i.e. they rotate the plane of polarization. In such liquids each molecule can be thought as a small crystal with an optical axis. If plane polarized light is sent to the molecule its polarization plane will be rotated.

Optical activity depends on the nature of the substance, the optical path length of the liquid column (l) in dm, the concentration (c) of the solution (g/100 mL), nature of the solvent, temperature (T) of the solution (Celsius degrees) and the wavelength (λ) of the light used, usually λ = 589 nm. The rotation is proportional to the inverse square of the wavelength. These parameters are considered in the Specific Rotation quantity given by Equation (1). To test our hypothesis, we have used fructose [8,9] and glucose [8,9]. Former substance shows a large Specific Rotation, [α]_D_^20^ = −92 deg cm^3^/(g dm) [8].

Glucose (C_6_H_12_O_6_) [10] is a monosaccharide, which is the simplest unit of a carbohydrate. It is the basic unit of polysaccharides: starch, cellulose, and glycogen, and it is the most abundant and most important sugar molecule in nature. Glucose is an aldohexose because its formula contains six-carbon atoms with a functional group aldehyde, and it is able to form a five or six-atom ring (including the oxygen) producing a furanose or a pyranose. Its 3D configuration leads to two different stereoisomers, I and II, where I deviates the plane of polarized light towards the right (+) and the II to the left (−). Moreover, the position of the chiral carbon of the aldehyde group and its configuration, gives the glucose D and L, depending if the OH group position is in the right or in the left of the main structure. Finally, the position of the OH group in the C1 produces an additional classification (that leads to a different chemical behavior) known as alpha or beta monosaccharide. In this work, the used glucose was α-D(+)-glucopyranose, which is a colorless powder, with molecular weight of 180.16 g/mol, solubility in water of 500 g/L and melting point of 146°C. Fructose (C_6_H_12_O_6_) is also a monosaccharide and a ketohexose, colorless powder and together with glucose makes the sucrose or common sugar. In this work, the β-D(−)-fructofuranose was employed. Solubility of fructose is 3.99 g/L at 20 °C and its melting point is lower than the one of glucose.

The optical characterization study of fructose that we performed, besides its optical activity, included the behavior of refractive index as a function of solutions of fructose and distilled water. Refractive index measurements were done with an Abbe refractometer. Results showing the behavior of the refractive index measurements are shown in in Section 4 along with the calibration plots. We notice a steady increment of the refractive index as fructose increases in the solution of fructose–water. These refractive index measurements have to be done once for each substance tested in order to build a standard curve. Regarding the glucose we prepared solutions with the same concentrations that for the fructose solutions and found that they presented almost the same refractive indices.

To investigate if linearly polarized light that passed through the substances was not affected in its linear state of polarization, except for the rotation of the plane of polarization, the following set up was used. A sodium lamp (λ = 589 nm) was used as light source with a polarizer in front of it. Then light passed through a glass cell 7 cm long that contained the solution. At the end a second polarizer (analyzer) measured the light state. The liquids study was made by examining the extinction light angle by sight. All lights in the laboratory were turned off to have a good adaptation of the eye to darkness. For the liquid samples tested just a rotation of the plane of polarization was present. No elliptical polarized light was noticed.

## 3. Interferometric Optical Configuration and Optical Principle

**Figure 1:**
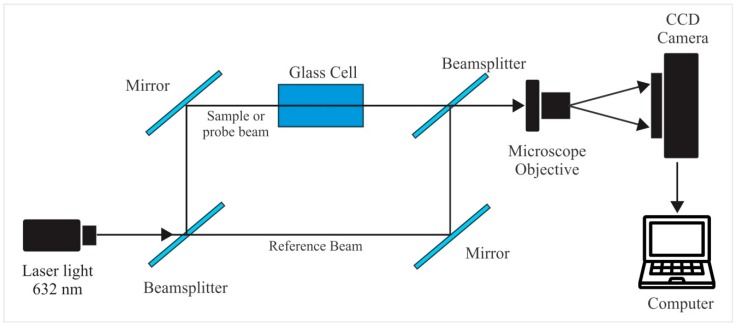
Mach–Zehnder interferometer.

The optical configuration that we suggest for an interference polarimeter is based on a Mach-Zehnder interferometer [11], Figure 1. The beamsplitters are of the absorption type. Reference and sample beams had normalized intensities of 0.714 and 0.286, respectively. Linearly polarized light comes from a He-Ne laser (polarization ratio 500:1) with a wavelength of λ = 633 nm. Beam cross intensity profile has a Gaussian shape. We can call trajectory 1 the “reference” wave and trajectory 2 the “sample” wave. Both plane waves interfere and because polarization of both beams is linear, parallel to each other and perpendicular to the plane of incidence, the interference pattern will consist of sinusoidal modulated fringes. These patterns are captured by a CCD camera. The interference pattern is modulated by the beam Gaussian intensity profile. Next we will describe theoretically the modifications of the interference pattern when the plane of polarization of the sample beam rotates.

**Figure 2:**
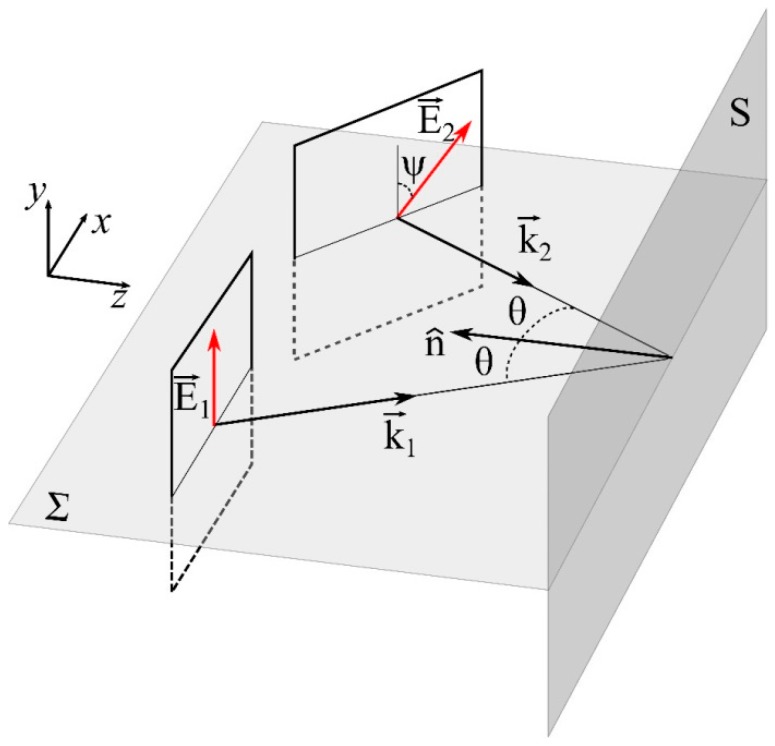
Superposition of two light beams with linear polarization and different angular positions.

At the interferometer output, two mutually coherent light beams, linearly polarized, with plane wavefronts are superimposed on an observation screen S parallel to the *xy* plane located at *z* = 0, Figure 2. The reference beam with its electric field ${\vec{E}}_{1}\left(\vec{r},t\right)$ is perpendicular to the incidence plane Σ, which is parallel to the *xz* plane. On the other hand the sample beam has its electric field ${\vec{E}}_{2}\left(\vec{r},t\right)$ subtending an angle ψ with the reference beam. Incidence angles are symmetric ($\theta =\theta_{1}=\theta_{2}$) with respect to the normal $\hat{n}$ of S.

Thus, assuming equal Gaussian amplitude distribution,

(2) $${\vec{E}}_{1}\left(\vec{r},t\right)={\vec{E}}_{01}\exp\left[j\left({\vec{k}}_{1}\cdot \vec{r}-\omega t+\varphi_{1}\right)\right]$$

(3) $${\vec{E}}_{2}\left(\vec{r},t\right)={\vec{E}}_{02}\exp\left[j\left({\vec{k}}_{2}\cdot \vec{r}-\omega t+\varphi_{2}\right)\right]$$

where:

(4) $${\vec{E}}_{01}=E_{0}\exp\left(-\frac{r_{0}^{2}}{w_{0}^{2}}\right)\hat{ȷ}$$

(5) $${\vec{E}}_{02}=E_{0}\exp\left(-\frac{r_{0}^{2}}{w_{0}^{2}}\right)\left[\cos\theta \sin\psi \hat{ı}+\cos\psi \hat{ȷ}+\sin\theta \sin\psi \hat{k}\right]$$

$r_{0}$ is the radial distance from the center of the Gaussian spot over the plane wavefront, $w_{0}$ is the radius at which the electric field amplitude falls to 1/e, and

(6) $${\vec{k}}_{1}\cdot \vec{r}=k\left(x\sin\theta +z\cos\theta \right)$$

(7) $${\vec{k}}_{2}\cdot \vec{r}=k\left(-x\sin\theta +z\cos\theta \right)$$

where k=2π/λ is the wavenumber and λ is the wavelength. In an amplitude division interferometer, the output beams are emitted by the same source (φ1=φ2=0). Then, the resulting interference pattern is described by

(8) $$I\left(x,y\right)=\left[I_{1}+I_{2}+2\sqrt{I_{1}I_{2}}\cos\psi \cos\left(2kx\sin\theta \right)\right]\exp\left(-\frac{2r_{0}^{2}}{w_{0}^{2}}\right)$$

where $I_{1,2}=\frac{1}{2}\varepsilon_{0}cE_{01,02}^{2}$, and the visibility of interference fringes is found to be

(9) $$V=\frac{I_{max}-I_{min}}{I_{max}+I_{min}}=\frac{2\sqrt{I_{1}I_{2}}\cos\psi }{I_{1}+I_{2}},\;0\leq \psi \leq 90{}^{\circ}$$

**Figure 3:**
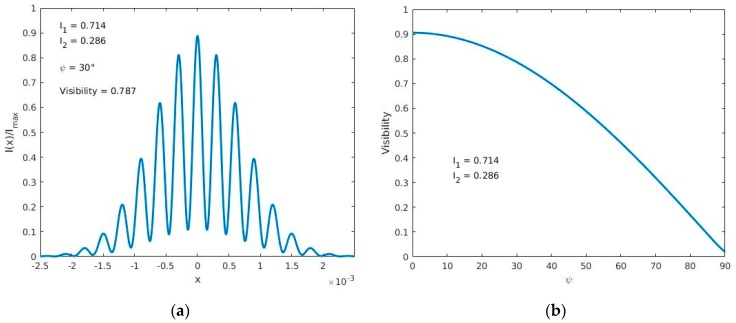
(**a**) Theoretical cross section Intensity pattern as a function of length of an interference pattern; (**b**) Theoretical behavior of interference pattern visibility as a function of the rotation angle (degrees) of the sample plane polarized light.

A theoretical plot showing the interference pattern intensity profile, for an angle ψ, is shown in Figure 3a. By measuring the maximum intensity and the adjacent minimum intensity of different patterns, like the one shown in Figure 3a, we can get the visibility for each angle of rotation ψ. Plot in Figure 3b shows this behavior.

## 4. Results of Experimental Studies

To find the calibration plots of Visibility as a function of solutions concentrations a glass cell, 7 cm long, was inserted in the “sample” light trajectory of the Mach-Zehnder interferometer, Figure 1. Solutions with different mixing ratios of fructose-water or glucose-water were considered. Table 1 shows the concentration of each sample.

**Figure 4:**
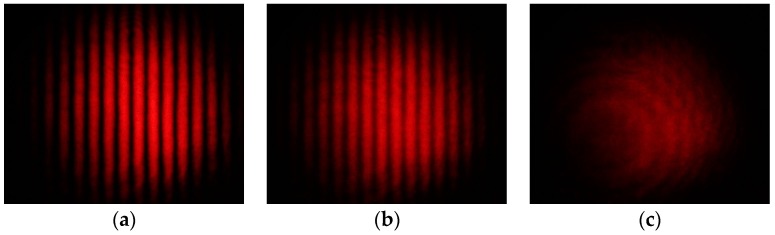
Photographs of interference patterns. The parameter was the concentration of fructose-water solutions. Note that the fringes visibility decreases because as concentrations increases the rotation angle of linearly polarized light increases. Amount of fructose in the solution was: (**a**) no fructose; (**b**) 4.2 g; and (**c**) 7.8 g.

Each amount of fructose or glucose was mixed in 4 mL of distilled water. Solutions were poured in the cell, one at a time, and photographs of the interference patterns were taken. Some of these interference patterns are shown in Figure 4. Experiments were performed at room temperature.

It should be mentioned that when solutions have a low concentration of fructose they were poured in the cell and after a few minutes the interference pattern was steady. However, for heavy solutions we had to wait some minutes so that the mixture stabilizes. It seems some “currents” in the bulk of the liquid were present. We had to stir lightly the solution to mix it well. Then photographs of the interference patterns were taken and they were investigated with the following method.

**Figure 5:**
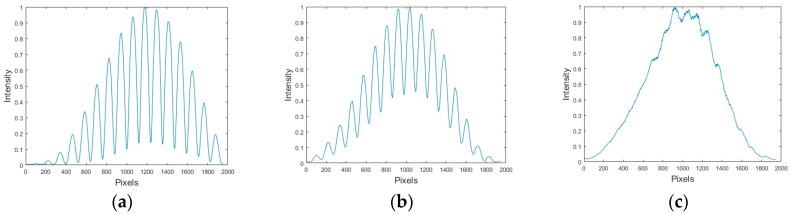
Intensity profiles as a function of pixels (length) obtained through a computer analysis of the interference patterns shown in Figure 4; (**a**) Intensity vs pixel number; (**b**) Intensity vs pixel number explanation; and (**c**) Intensity vs pixel number explanation.

In order to calculate the fringes Visibility of the interference patterns photographs were recorded and analyzed with a computer. The images had a resolution in pixels of 3456 × 2304 and captured in color. However, the intensity values were converted to gray levels ranging from 0 to 255. Some samples (sub-images) were taken to perform the analysis. In each photograph a region of interest was considered. The region consisted of a horizontal strip that passed through the center of the image. Then to avoid noise a low-pass filter was applied to each column. This gave us a one-dimensional vector of size equal to the width of the image with average values. This vector can be considered as a typical cross-section that passes through the center of the image. Some plots of this vector are shown in Figure 5. The abscissa axis represents pixels, and the ordinate axis the intensity values.

**Figure 6:**
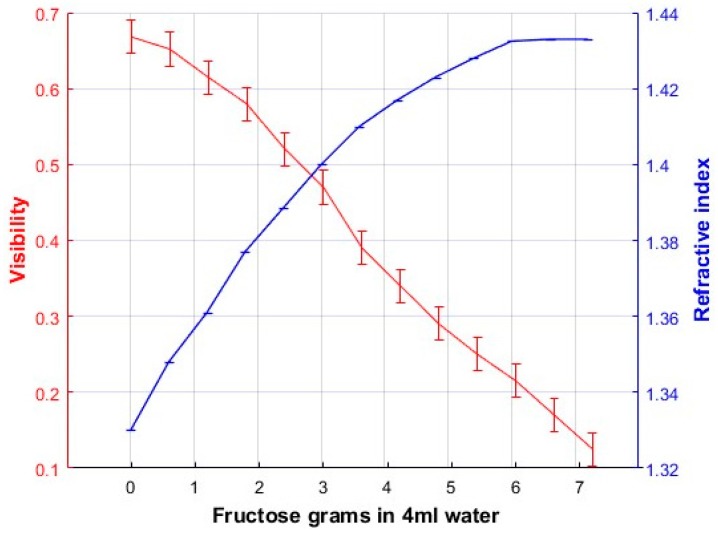
Calibration plot when solutions of fructose-water were considered.

**Figure 7:**
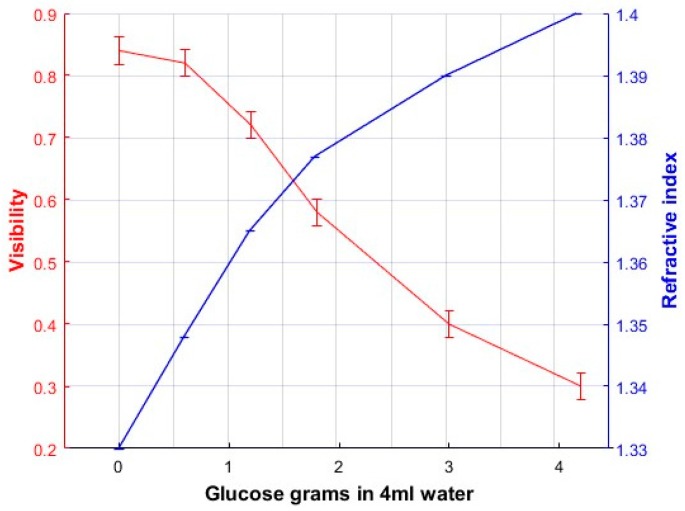
Calibration plot when solutions of glucose-water were considered.

From the plots shown in Figure 5 we can calculate the visibility for each graph by taking the maximum intensity value and the adjacent minimum. These visibility values were plotted as a function of the amount of fructose or glucose concentration in the solution and the refractive index of the solutions described in Section 2, and they are presented in Figure 6 and Figure 7. These are the calibration plots. Thus, if one needs to know the concentration and/or the refractive index of a fructose or glucose-water solution, a portion of it could be poured in the cell and through the Visibility plot of the interference patterns (the calibration plot) the concentration and refractive index could be known. The length of error bars in the visibility axis of Figure 6 and Figure 7 was calculated considering that the minimum difference of intensity measurements in the plots of Figure 6 was 0.008. Due to the use of a good balance to weight the amount of fructose/glucose, and the good instrument to measure the amount of water, the uncertainty in the concentration axis was assumed to be negligible.

Regarding the sensitivity, dynamic range and resolution [12] of the polarimeter we have done the following calculations. Plots in Figure 6 and Figure 7 have been considered. Figure 6 shows the calibration plot when fructose has been used. To calculate the sensitivity two points in the plot were chosen in the region where the plot shows a linear behavior. Saturation region has not been considered. Points have the following coordinates (1.8, 0.58) and (5.4, 0.25). The slope is found to be – 0.0916 (−5.23°). For the plot in Figure 7 that considers glucose the points have the coordinates (1.2, 0.7) and (3, 0.45). The slope is found to be −0.166 (−9.45°). The angles are taken from the abscissa axis. This sensitivity values represents the change in visibility as a function of the change in liquids concentration.

From the same plots in Figure 6 and Figure 7 we can get the dynamic ranges. That is the difference between the smallest and largest concentrations that can be measured. For the fructose we can get a dynamic range of 3.6 g/4 mL (9.22 g/10 mL). For the glucose the dynamic range is 2.59 g/4 mL (6.25 g/10 mL).

To find out the concentration resolution we use the following process. In the plot of Figure 6 (fructose) we choose that the minimum change in visibility that we can distinguish is 0.01. Then we can distinguish a change in concentration of 150 mg/4 mL (37.5 mg/mL). This is the resolution in concentration. Regarding the calibration plot for the glucose, Figure 7, we can distinguish a minimum change of visibility of 0.013. This will give us a change in concentration of 90 mg/4 mL (22.5 mg/mL).

Having calculated the dynamic range and the resolution for glucose we can compare them with other published work. This is shown in Table 2. Reference [13] mentions a method based on the effect of Surface-Plasmon Resonance (SPR) with a common-path heterodyne interferometry. Reference [14] shows an interferometric system with a measuring length host. Although these systems show better resolution their dynamic range is very short and besides this, the optical and electronic complexity, and cost, is very high.

In order to know the concentration, a polynomial plot should be fitted to the experimental data. For example, for fructose, Figure 6, we have fitted a fourth order polynomial of the type

(10) $$V\left(c\right)=a_{0}+a_{2}c^{2}+a_{3}c^{4}.$$

With the following result:

(11) $$V\left(c\right)=0.643-0.31c^{2}+0.048c^{4}$$

**Figure 8:**
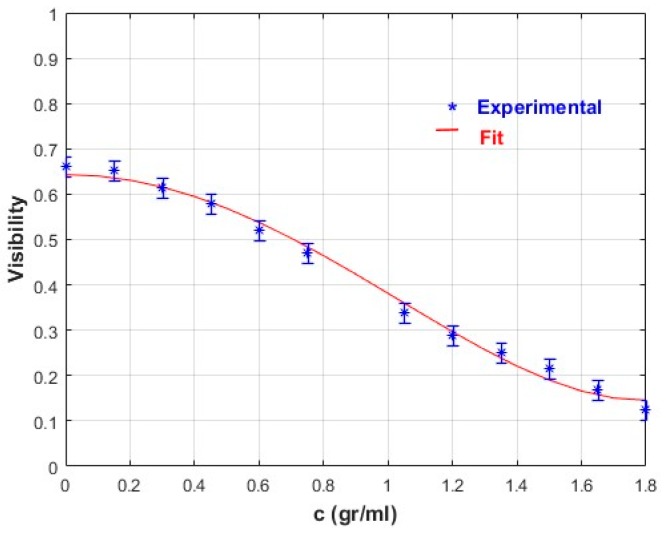
Fourth order polynomial fitted to the experimental points (Fructose).

Equation (11) has been plotted along with the experimental data and is shown in Figure 8.

The unknown solute concentration can be retrieved using the equation

(12) $$c\left(V_{m}\right)=\sqrt{\frac{-a_{2}-\sqrt{a_{2}^{2}-4a_{3}\left(a_{0}-V_{m}\right)}}{2a_{3}}}$$

where *V_m_* is the measured visibility. The polynomial function in Equation (10), which is a fourth order approximation of the cosine function when a Taylor series is used, is proposed to predict visibility measurements as a function of solute concentration. The correlation coefficient between experimental and predicted visibility was R = 0.994.

## 5. Conclusions

An amplitude division interferometer has been used to assess the concentration of solutions by measuring the visibility of interference fringes. This novel wavefront division polarimeter, of the Mach–Zehnder type, comprises common optical elements in an optical laboratory and an image acquisition device. Calibration plot and digital image processing are key features in the proposed system. The selection of an optimally biased visibility value should allow the measurement of unknown (left) levo- as well as dextro-rotatory substance concentrations. In order to increase the sensitivity, spatial frequency in the interference pattern could be decreased keeping at least one full cycle into the camera view field with the aim of achieving less uncertainty in the visibility measurement.

As examples of studied solutes we have used fructose and glucose. These substances dissolved in water only rotate the plane of polarization. They do not produce elliptical polarization. We conclude that the described method is versatile because it can be adapted to the measurement of substances with different optical activity. For example, if a substance shows low optical activity, the length of the cell can be made longer to have better sensitivity. The opposite can be made when solutions with high optical activity are present.

## Figures and Tables

**Table 1:** Amount of fructose/ glucose in solutions containing 4 mL of distilled water.

Solution Number	Fructose or Glucose (g)
1	0.6
2	1.2
3	1.8
4	2.4
5	3
6	3.6
7	4.2
8	4.8
9	5.4
10	6

**Table 2:** Comparison of Dynamic Ranges and Resolutions with other published works when glucose was considered.

Reference	Dynamic Range	Resolution
Ref. [13]	45 mg/10 mL	0.141 mg/10 mL
Ref. [14]	200 mg/10 mL	48 mg/10 mL
This paper	6250 mg/10 mL	225 mg/10 mL
